# Structural Integrity of the Contralesional Hemisphere Predicts Cognitive Impairment in Ischemic Stroke at Three Months

**DOI:** 10.1371/journal.pone.0086119

**Published:** 2014-01-24

**Authors:** Rosalia Dacosta-Aguayo, Manuel Graña, Marina Fernández-Andújar, Elena López-Cancio, Cynthia Cáceres, Núria Bargalló, Maite Barrios, Immaculada Clemente, Pere Toran Monserrat, Maite Alzamora Sas, Antoni Dávalos, Tibor Auer, Maria Mataró

**Affiliations:** 1 Department of Psychiatry and Clinical Psychobiology, University of Barcelona, Barcelona, Spain; 2 Group of Computational Intelligence, University of the Basque Country UPV/EHU, San Sebastian, Spain; 3 Department of Neurosciences, Hospital Germans Trias i Pujol, Universitat Autònoma de Barcelona, Barcelona, Spain; 4 Institute for Brain, Cognition and Behaviour (IR3C), Barcelona, Spain; 5 Diagnostic Center for Image, Clinic Hospital, Barcelona, Spain; 6 Imatge Platform of IDIBAPS, Barcelona, Spain; 7 Department of Methodology of Behavioral Sciences, University of Barcelona, Spain; 8 MRC Cognition and Brain Sciences Unit, Cambridge, England; 9 Primary Healthcare Research Support Unit Metropolitana Nord, Institut Universitari d’Investigació en Atenció Primària (−IDIAP) Jordi Gol, Santa Coloma de Gramenet, Spain; National University of Singapore, Singapore

## Abstract

After stroke, white matter integrity can be affected both locally and distally to the primary lesion location. It has been shown that tract disruption in mirror’s regions of the contralateral hemisphere is associated with degree of functional impairment. Fourteen patients suffering right hemispheric focal stroke (S) and eighteen healthy controls (HC) underwent Diffusion Weighted Imaging (DWI) and neuropsychological assessment. The stroke patient group was divided into poor (SP; n = 8) and good (SG; n = 6) cognitive recovery groups according to their cognitive improvement from the acute phase (72 hours after stroke) to the subacute phase (3 months post-stroke). Whole-brain DWI data analysis was performed by computing Diffusion Tensor Imaging (DTI) followed by Tract Based Spatial Statistics (TBSS). Assessment of effects was obtained computing the correlation of the projections on TBSS skeleton of Fractional Anisotropy (FA) and Radial Diffusivity (RD) with cognitive test results. Significant decrease of FA was found only in right brain anatomical areas for the S group when compared to the HC group. Analyzed separately, stroke patients with poor cognitive recovery showed additional significant FA decrease in several left hemisphere regions; whereas SG patients showed significant decrease only in the left genu of corpus callosum when compared to the HC. For the SG group, whole brain analysis revealed significant correlation between the performance in the Semantic Fluency test and the FA in the right hemisphere as well as between the performance in the Grooved Pegboard Test (GPT) and theTrail Making Test-part A and the FA in the left hemisphere. For the SP group, correlation analysis revealed significant correlation between the performance in the GPT and the FA in the right hemisphere.

## Introduction

Stroke and cerebrovascular disease is a major cause of mortality and disability worldwide [1]. Approximately 64% of patients who have experienced stroke have some degree of cognitive impairment and up to a third of them develop dementia [2].

With cerebral ischemia, reductions in cerebral blood flow disrupt energy metabolism resulting in perturbation of ionic pumps and disruption of ionic homeostasis [3]. Cytotoxic edema occurrence produces both a decrease in the extracellular volume fraction and changes in membrane permeability [4]. These events reduce white matter (WM) integrity locally, at the primary lesion location, due to tissue damage or remotely as a consequence of anterograde Wallerian (WD) and/or retrograde axonal degeneration [5]. Furthermore, reduced WM integrity has been found to be associated with cognitive impairment [6], [7], [8].

The role of the non-injured hemisphere in stroke recovery is still controversial. Some imaging studies suggest that contralesional functional networks are significantly involved in post-stroke functional recovery [33], [71]; although the interpretation of the results regarding to their positive or negative implication in patient’s recovery is disputed (for a review of the literature see 71). At a structural level, some diffusion tensor imaging (DTI) studies report increased anisotropy in the contralesional hemisphere (e.g. thalamus) after stroke [24], [25], structural remodeling in ipsilateral and contralesional corticospinal tracts [25] and changes in the number of neural pathways in areas both ipsilateral and contralateral to the stroke [34]. Studies of tract’s fractional anisotropy (FA) asymmetries have concluded that the contralesional corticospinal tract may play a role in motor recovery after unilateral stroke [72], [73], [74], [23].

Animal studies of ischemic stroke often use FA to study the temporal evolution of WM changes [9], [10], [11], [12]. From these studies it has been established that disruption of the brain tissue microstructure results in a significant reduction in the FA during the subacute and chronic phases of cerebral ischemia. Human studies usually use FA [13], [14], [15], [16] and Mean Diffusivity (MD) [17], [18], [19] to study the recovery of the injured brain over time. Regarding FA values, microstructural integrity of normal-appearing WM improves during 1 and 2 years following ischemic stroke, achieving subsequent stabilization [14].

Animal studies suggest that structural remodeling of WM in both the ipsilesional and contralesional hemispheres plays a role in motor recovery [20], [21], [22], [23]. On the other hand, DTI studies of stroke patients have demonstrated that reduced contralesional WM integrity of the corticospinal tract in chronic stroke [24] is associated with poorer motor skill recovery [25] in stroke patients compared to patients with better motor recovery and healthy controls (HC) [25]. In these studies, larger interhemispheric asymmetries in FA for this anatomical region have been associated with reduced motor recovery [26], [27], [28], [29], reduced skill improvement in response to training [30] as well as motor dysfunction after stroke [27], [31]. Although plastic changes in the contralesional hemisphere have already proved to play a role in stroke recovery [32], [33], [25], [34], this role remains still unclear from a cognitive point of view.

Prior work suggests that DTI may provide information about different pathophysiological processes and may be one of the most sensitive neuroimaging biomarkers of vascular damage [35], [36], [37], [8]. Although, FA is the most widely studied diffusion metric, there is a growing interest in investigating WM microstructural mechanism underlying the FA change by analyzing other – more direct – diffusion metrics, such as axial (AD) and radial (RD) diffusivity. A recent study identified RD and AD decrease accompanying FA increase in areas surrounding the anterior cingulate cortex after 4-week integrative body-mind training, a form of mindfulness meditation [38]. Reduction in RD is usually interpreted as improved myelination, whereas reduction in AD is associated with axon morphological changes, such as changes in axonal density or caliber [39], [40].

The objectives of the study in this paper were: 1) to evaluate the effects of right hemispheric stroke on left hemispheric WM; 2) to investigate the microstructural mechanism underlying the eventual WM change 3) to assess their effect on cognitive recovery of stroke patients at three months after stroke. We test the following hypothesis: a) Stroke patients with poor cognitive recovery will show more widespread WM disruption in the contralesional (left) hemisphere; b) Stroke patients with good cognitive recovery will have better contralesional FA integrity; c) The better contralesional WM integrity will be related to the cognitive performance of stroke patients with good cognitive recovery. To address these questions we used a hypothesis-driven TBSS approach.

## Materials and Methods

### 1.1. Subjects

This is a prospective and longitudinal study that included a group of consecutive ischemic stroke patients (S) admitted at the Stroke Unit at the Germans Trias i Pujol Hospital from August 2008 to May 2012 and a group of healthy controls (HC). Stroke patients underwent a complete neuropsychological assessment in the acute phase (first 72 hours), and another at three months when the DTI study was performed. In the control group, cognitive assessment and DTI were performed in the same day.

Potential participants were included in the study if they had 1) First territorial ischemic stroke in the territory of middle, anterior or posterior cerebral arteries (MCA, ACA, PCA); 2) modified Rankin Scale (mRS) score of 0 and Barthel score of 100 before admission; 3) Age in the range between 40 and 75 years; 4) Absence of severe aphasia (fourteenth scoring item of National Institute of Health Stroke Scale (NIHSS) ≤1); 5) Absence of alcohol or drug abuse, psychiatric comorbidities, or severe visual or hearing loss; 6) Absence of contraindications to undergo MRI or severe claustrophobia.

From the 30 patients included in the study, we chose patients with right hemisphere stroke (n = 17), from which 3 were discarded due to acquisition problems (n = 1), and because the lesion volume fell outside 1.5 times the interquartile range (n = 2). The final study sample comprised 14 patients.

Eighteen healthy volunteers from the Barcelona-Asymptomatic Intracranial Atherosclerosis (AsIA) study [41], [42] matched by age, sex, education, and handedness with the stroke patients were recruited as the control group. None of them had a previous history of neurological or psychiatric diseases and brain scans were reported as normal. Information on demographical characteristics and vascular risk factors were collected in each patient based on their medical history.

The study was approved by the institutional ethics committee (Comissió de Bioètica de la Universitat de Barcelona (CBUB); Institutional Review Board (IRB) 00003099 Assurance number: FWA00004225; http://www.edu/recerca/comissiobioetica.htm and the research was conducted in accordance with the Helsinki Declaration. Written informed consent was obtained from each participant prior to taking part in the study.

### 1.2. Measurement of Cognitive Function and Grouping Criteria

Information about previous cognitive impairment was collected by a trained neuropsychologist with the short version of the Spanish Informant Questionnaire on Cognitive Decline in the Elderly (S-IQCODE) [43] and the Frontal Behavioral Inventory (FBI) [44] on admission day. Premorbid Intelligence was estimated using the vocabulary subtest of Wechsler Adults Intelligence Scale (WAIS-III-R) [45] at three months post-stroke.

Patients underwent two neuropsychological examinations at different times. First examination was performed within the first 72 hours after the stroke. We selected a test battery that covered a variety of possible cognitive manifestations of vascular brain injury. Attentional abilities were explored by the Digit Span Forward Test (WAIS-III-R) [45], the subtest of attention extracted from de Montreal Cognitive Test (MOCA) [46], and the Line Cancellation Test (LCT) [47]. Executive abilities were assessed with the Digit Span Backwards from WAIS-III-R) [48], part B of the Trail Making Test [47], Phonological fluency (letter P) [47], and Semantic fluency (animals) [47]. Language abilities were assessed with spontaneous speech (talking briefly about his/her health problems), repetition, understanding items extracted from The Mental Status Examination in Neurology [48], writing of one sentence, item extracted from the Mini Mental State Examination Test (MMSE) [49], and naming with the short version (15-items) of the Boston Naming Test [50]. Premotor abilities were assessed with Luria’s sequences test, Rhythms subtest extracted from the MOCA test [46], and interference and inhibitory control subtest extracted from the Frontal Assessment Battery [51]. Speed and visuomotor coordination were assessed with the part A of the Trail Making Test [47] and the Grooved Pegboard Test (GPT) [52]. Neuropsychological examinations also included the MMSE [49], as a global cognitive test and the Geriatric Depression Scale (GDS) [53]. The acute neuropsychological examination was performed in a fixed order that took approximately 60 minutes to complete. If the patient was fatigued, the testing was split between two sessions carried out in the same day.

Second cognitive examination took place at three months after stroke lasting at most 2 hours. It should be mentioned that for this study we only considered the tests that had been used in both examinations. HC received a similar neuropsychological assessment to S at the acute phase. Stroke patients were dichotomized into two subgroups according to their level of cognitive recovery between acute and subacute phase. First, a paired t-test was conducted to determine the cognitive tests in which patients had significantly improved. Second, subjects were considered to demonstrate a good cognitive recovery (SG group) if they had normalized or improved 1.5 SD in at least three of these tests.

Statistical analyses were performed with the Statistical Package for the Social Sciences (SPSS Inc., Chicago, IL, USA), version 17.0 for Windows. Baseline characteristics were summarized as mean ± standard deviation (SD) for continuous variables and proportions (n, %) for categorical variables. The threshold for statistical significance was set at P<0.05.

### 1.3. MRI Acquisition and Lesion Analysis

All images were acquired at a 3T Siemens Magneto TIM Trio (Siemens Diagnostics Healthcare, Erlangen, Germany) at the Image Platform of IDIBAPS, Centre de diagnostic per la Imatge from Hospital Clínic (CDIC), Barcelona. We used a 32-channel phased-array head coil with foam padding and head phones to restrict head motion and suppress scanner noise. The MRI protocol included a set of magnetization prepared rapid gradient echo (MP-RAGE) T1-weighted images (repetition time [TR]: 2300 ms; echo time [TE]: 3 ms; flip angle: 15°; field of view: 245 mm; and voxel size: 1×1×1 mm^3^) and two runs of DWI. DWI was acquired in 30 non collinear diffusion directions, with a b-value of 1.000 s/mm^2^ and one with a value of 0 s/mm^2^, with the following echo planar acquisition protocol: [TR]: 9300 mm; [TE]: 94 ms; flip angle, 15°; field of view: 240 mm; no gap; and voxel size: 2×2×2 mm^3^.

Infarct depth (cortical, subcortical or both), laterality (left/right) and vascular territory involved were determined in the first 24 hours employing Computed Tomography (CT) and/or Magnetic Resonance (MRI). Lesion volume was calculated in the subacute phase. T2-weighted images (TR: 5520 ms; echo time [TE]: 94 ms) and fluid attenuated inversion recovery images (FLAIR; [TR]: 9040 ms; [TE]: 85 ms; inversion time [TI]: 2500 ms; and voxel size: 0.86×0.86×6.5 mm^3^) were collected and analyzed by a trained neurologist (M.M). Lesion volume was determined using the three largest diameters along the three orthogonal axes divided with 2 (AxBxC/2) [54].

### 1.4. Image Pre-processing

DWI pre-processing included motion and eddy current correction using FSL’s Eddy Correct Tool using the FMRIB Diffusion Toolbox (FDT) (Analysis Group, FMRIB, Oxford, UK). In order to eliminate spurious voxels, skull stripping of the T2 weighted b = 0 volume was achieved using FSL’s Brain Extraction Tool (BET) [55], and was used as a brain-mask for all other diffusion maps. The second DWI run was linearly co-registered (FLIRT) to the first, and the two runs have been averaged. FMRIB's Diffusion Toolbox - FDT v2.0 was used for the tensor modeling of the diffusion parameters to produce DTI data. Microstructural maps of fractional anisotropy (FA) and mean diffusivity (MD) were entered into group analysis using Tract Based Spatial Statistics - TBSS v1.2 [56] which is part of FSL data processing suite [57].

### 1.5. Diffusion Tensor Image Group Analysis

#### Tract-Based Spatial Statistics (TBSS)

All subjects' FA data were aligned into a common space using the nonlinear registration tool FNIRT [58], [59], which uses a b-spline representation of the registration warp field [60], [61], resulting in all images transformed into 1 mm isotropic, MNI152 standard space. Next, all participants’ FA volumes were averaged and a mean FA skeleton was created from all voxels with a FA threshold = 0.2 to reduce inclusion of voxels that are likely composed of multiple tissue types or fiber orientations. Each participants’ aligned, standard space, FA maps were then projected onto this skeleton to create a 4D skeletonized volume (3D skeletal volume × number of subjects) which was then fed into voxelwise group statistics.

### 1.6. Statistical Analysis of the DTI Data

Randomize tool (v2.1; www.fmrib.ox.ac.uk/fsl/randomise/index.html) from the FMRIB software library with a number of permutation tests set to 5000, was applied on the FA maps [62. 56], to identify clusters of voxels that were significantly different between the HC and the S, SP, and SG groups. Significant clusters were identified using the Threshold-Free Cluster Enhancement (TFCE) choosing a more restrictive threshold at a p-value ≤0.02 corrected for Family Wise Error (FWE) via Gaussian Random Field theory [63].

Other diffusion-derived data (RD and AD) projections on the TBSS skeleton were also calculated for each subject. The spatial normalization transformations computed for the FA maps were applied on the RD and AD maps to achieve their nonlinear registration, which was projected on the TBSS skeleton. The resulting 4D volumes were also used for voxelwise cross-subject statistics.

The following statistical comparisons were made for each TBSS diffusion map: 1) Whole-brain analysis between HC and S subgroups; 2) Whole-brain ANCOVA analysis with the selected cognitive tests as covariates of interest to study differences between HC, SG, and SP; 3) Spearman’s correlations analysis between the selected cognitive tests as covariates of interest and the TBSS diffusion maps.

## Results

### 3.1. Sample Characteristics

Demographic and clinical data are shown in Table 1. All subjects were right handed (mean = 96.91±10.08) except for one ambidextrous subject. There were no significant between-groups differences regarding to premorbid IQ, sex, gender and elapsed time between stroke onset and neuropsychological assessment in the acute phase (data not shown). Only a higher frequency of diabetes mellitus was found in the stroke group with poor cognitive recovery compared to the HC group. Table 2 shows stroke severity at baseline of the National Institute of Health Stroke Scale (NIHSS) and characteristics of the ischemic lesions (location, brain hemisphere, volume and vascular territory). All infarcts were in the territory supplied by the right MCA with the exception of 2 infarcts located in the right PCA territory. There were no significant between-group differences either regarding to the volume of the lesion (t_12_ = −0.524; p = 0.610), neurological severity, measured with the NIHSS (t_6.284_ = 1.272; p = 0.248) at baseline and at three months (Z = 0.000; p = 1.000), functional status, measured with the Barthel Scale at 3 months (Z = −0.091; p = 0.928) and the treatment received (all patients received mechanical Thrombectomy, with the exception of two patients, who received fibrinolytic treatment with rt-PA) (data not shown).

**Table 1:** Demographical and Clinical data.

	HC (n = 18)	SG (n = 6)	SP (n = 8)	HC - SG	HC - SP	SG - SP
**Sociodemographic Factors**				**p**	**p**	**p**
Age (years)	63 (60.75–67)	58.67±11.021	66.50±6.370	0.867	0.155	0.172
Women	7 (38.9%)	1 (16.7%)	2 (25%)	0.319	0.413	0.615
Education (years)	7.33±4.087	9.50±3.728	8±5.904	0.264	0.741	0.597
Vocabulary subtest	37.78±7.856	36.83±12.024	33.86±10.976	0.825	0.326	0.650
Edinburgh Test	95.56±13.492	96.67±5.164	99.38±1.768	0.452	0.723	0.280
**Vascular Risk Factors**						
Hypertension	8 (44.4%)	3 (50%)	5 (62.5%)	0.590	0.223	0.413
Dyslipidemia	9 (50%)	3 (50%)	4 (50%)	0.680	0.664	0.704
Diabetes Mellitus	1 (5.6%)	2 (33.3%)	4 (50%)	0.143	**0.020**	0.471
Smoking	6 (33.3%)	1 (16.7%)	2 (25%)	0.414	0.607	0.563
Alcohol intake	9 (50%)	1 (16.7%)	4 (50%)	0.171	0.664	0.238

Values are means ± standard deviations in Student’s t-test or medians (interquartile range) in Mann-Whitney test for continuous variables. Values are n (%) for categorical variables in Chi-square test and Fisher’s exact test. p shows statistical comparison between groups.

**Table 2:** Stroke characteristics and neurological impairment at baseline.

Patients	Baseline NIHSS	Hemisphere	Infarct location	Lesion Volume (cm^3^)	Arterial Territory
STROKE GROUP WITH GOOD COGNITIVE RECOVERY					
**1**	1	R	Frontal	0.1	MCA_ACA
**2**	17	R	Basal Ganglia+Insula	8.2	MCA
**3**	4	R	Occipital Cortex+Centrum Semiovale	52.3	PCA
**4**	16	R	Lenticular	3.6	MCA
**5**	5	R	Fronto-Parietal+Premotor+Insula	4.6	MCA
**6**	7	R	Insula+Frontal inferior	14.5	MCA
STROKE GROUP WITH POOR COGNITIVE RECOVERY					
**7**	8	R	Temporo-Parietal posterior	15	MCA
**8**	7	R	Temporo-Occipital	20.9	PCA
**9**	7	R	Basal Ganglia+Corona Radiata	42	MCA_ACA
**10**	17	R	Lenticular+Fronto-Parietal	7.3	MCA
**11**	13	R	Temporo-Parietal+Intern Capsule	34	MCA
**12**	9	R	Fronto-Parietal+Insula	32.5	MCA
**13**	14	R	Basal Ganglia+Insula+Corona Radiata	36	MCA
**14**	9	R	Basal Ganglia+Corona Radiata	17.6	MCA

Abbreviations: NIHSS = National institute of Health Stroke Scale; R = right; MCA = Middle Cerebral Artery; PCA = Posterior Cerebral Artery; ACA = Anterior Cerebral Artery;

### 3.2. Neuropsychological Testing

Stroke groups in general demonstrated a significant acute-to-subacute improvement in the following cognitive tests: Mini-Mental State Examination (MMSE), Semantic Fluency (SF) (naming animals in one minute), Boston Naming Test (BNT), TMTA and the GPT (data not shown). The reader has to keep in mind that cognitive improvement corresponds to increasing score in the first three and decreased time to complete the last two tests. This fact is important for the interpretation of the correlation between FA and the test scores.

At the subacute phase, the SG group showed significant differences from the HC group only in the MMSE (Z = −2.417; p = 0.016). The SP group showed significant differences from the HC group in the following cognitive tests: MMSE (Z = −2.294; p = 0.022); Luria’ sequences test (Z = −2.704; p = 0.007); Interference and Inhibitory Control test (Z = −2.196; p = 0.028); Phonetic Fluency test (t_24_ = −2.661; p = 0.014); Trail Making Test part-A (t_7.485_ = −2.392; p = 0.046); Grooved Pegboard Test (Z = −2.613; p = 0.009) and the Attentional subtest (MoCA) (Z = −3.634; p = 0.000).

Regarding to the two stroke subgroups, we only found significant differences between SG and SP in the BNT (Z = −2.089; p = 0.037) (Table 3).

**Table 3:** Neuropsychological performance.

	HC (n = 18)	SG (n = 6)	SP (n = 8)	HC - SG	HC - SP	SG - SP
**General Cognitive Function**				**p**	**p**	**p**
MMSE	29.11±1.28	27.50±1.38	27.25±2.49	**0.016**	**0.022**	0.250
**Sustained Attention**						
MoCA subtest (/11)	11	10.83±0.41	9.50±1.77	0.083	**0.000**	0.072
Digit Span Forward (WAIS-III)	5±1.14	4.67±1.37	4.75±1.49	0.560	0.642	0.462
**Working memory**						
Digit Span Backwards (WAIS-III)	3.83±1.04	3.83±0.75	3.38±1.30	0.747	0.274	0.643
**Premotor functions**						
Luria' sequences (/5)	5	4.67±0.82	3.87±1.81	0.083	**0.007**	0.227
Rhythms subtest (/10)	8.78±1.35	7±2.45	6.75±3.11	0.054	0.060	0.713
Interference and Inhibitory control (/3)	2.89±0.32	2.83±0.41	2.38±0.74	0.727	**0.028**	0.762
**Verbal fluency**						
Letter (P)	11.67±3.11	12.17±4.67	7.50±4.81	0.814	**0.014**	0.522
Semantic (Animals)	16.39±3.74	16.67±6.62	13.50±4.69	0.898	0.106	0.467
**Language**						
Boston Naming Test (/15)	11±2	13 (10–14)	9±3.02	0.152	0.056	**0.037**
**Psychomotor speed ** ***(seconds)***						
Trail Making Test A (seconds)	59.94±23.45	54.50±22.189	127.50±84.34	0.968	**0.046**	0.064
Grooved Pegboard Test (preferred hand; seconds)	72.33±11.87	68.83±14.26	146.25±97.16	0.557	**0.009**	0.109
**Visuospatial Skills**						
Right Line cancellation test (/18)	18	18	18	–	–	–
Left Line cancellation test (/18)	18	18	18	–	–	–

Values are means ± standard deviations in Student’s t-test or medians (interquartile range) in Mann-Whitney test for continuous variables. Values are n (%) for categorical variables in Chi-square test and Fisher’s exact test. p shows statistical comparison between groups. Abbreviations: MMSE = Mini Mental State Examination; HC = Healthy Control; SG = Stroke Group with good recovery; SP = Stroke group with poor recovery.

### 3.3. Fractional Anisotropy

**Figure 1:**
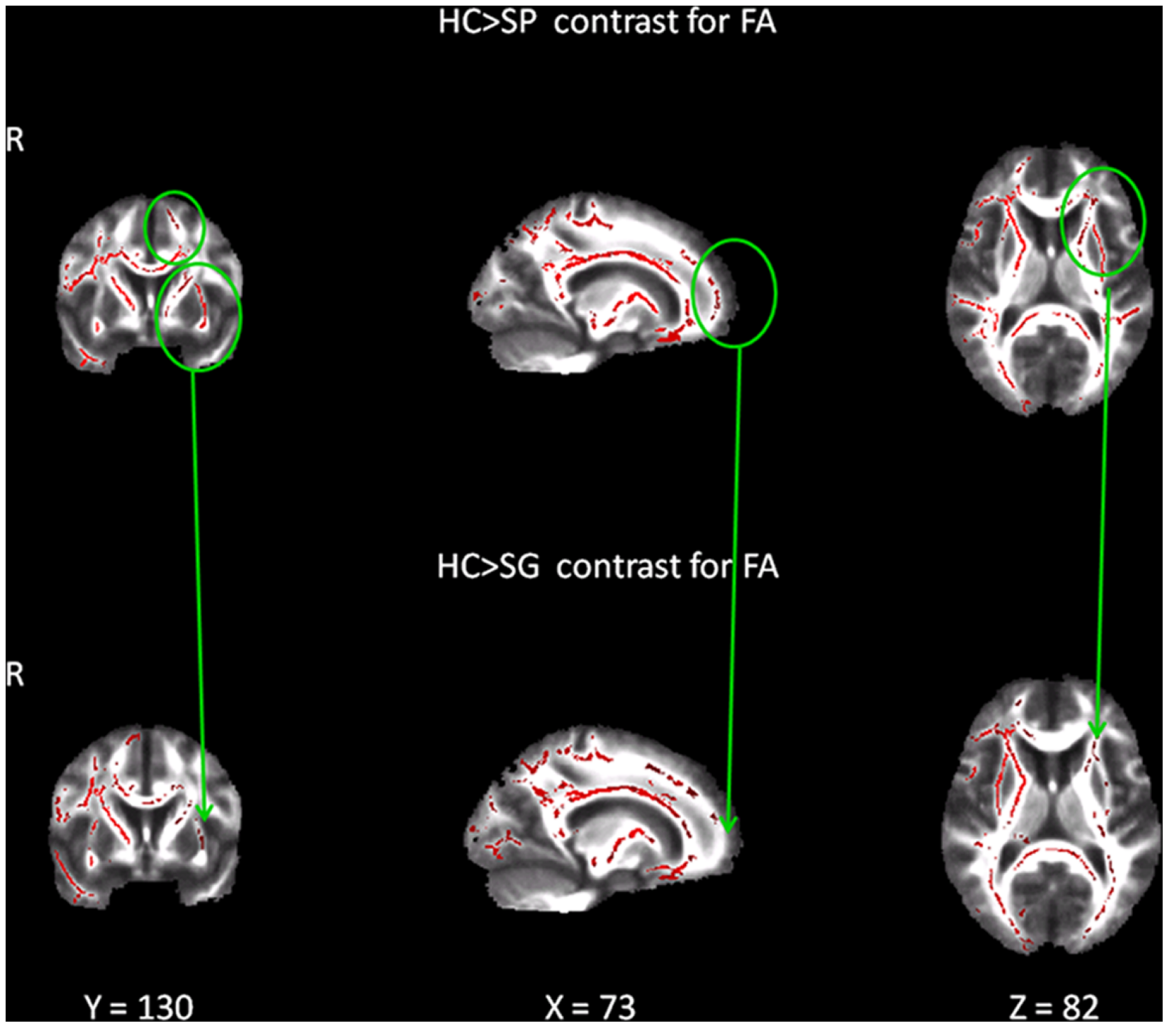
Significant changes in FA for the stroke group with poor recovery (SP) and the stroke group with good recovery (SG) at 3 months when compared with healthy controls (HC). The red color identifies clusters with significant decrease of FA. Statistical maps are represented in radiological convention (right corresponds to left hemisphere), superimposed on an MNI152 template. The threshold for significance was set at p≤0.02 corrected for multiple comparisons. Circles highlight the locus of clusters in the HC>SP contrasts not found in the HC> SG contrast.

For the whole stroke group (S), significant decrease of FA was found only in right hemisphere when compared to the HC (p = 0.001, d = 1.29) (Table 4). When analyzed separately, both SP and SG groups continue to show significant FA disruption in the right hemisphere (data not shown). SP group showed significant decrease of FA also in several anatomical areas of the left hemisphere (p = 0.02, d = 0.88); whereas SG group showed significant FA disruption only in one anatomical area of the left hemisphere (P = 0.02, d = 0.88) (Table 5, Figure 1).

**Table 4:** Clusters of significant FA, RD and AD differences between Stroke and Healthy Controls group.

Brain Lobe	Anatomical Region	Size (mm^3^)	MNI coordinates			Z-max	Z	p	d
			x	y	z				
	***Fractional Anisotropy***								
	**HC>S**								
BG	Thalamic Radiation (R)	755	77	119	65	1.00	3.09	0.001	1.299
BG	Corticospinal Tract (R)	483	72	118	75	1.00	3.09	0.001	1.299
T	Inferior Fronto_Occipital Fasciculus (R)	704	68	143	59	1.00	3.09	0.001	1.299
F	Uncinate Fasciculus (R)	138	56	129	58	1.00	3.09	0.001	1.299
BG	Anterior Limb of Internal Capsule (R)	202	77	121	68	1.00	3.09	0.001	1.299
BG	Retrolenticular part of Internal Capsule (R)	393	66	148	67	1.00	309	0.001	1.299
	***Radial Diffusivity***								
	**S>HC**								
BG	Anterior Limb of Internal Capsule (L)	493	105	142	68	0.99	2.88	0.002	1.21
BG	Anterior Limb of Internal Capsule (R)	521	77	121	69	1.00	2.88	0.002	1.21
BG	Retrolenticular part of Internal Capsule (L)	265	126	101	69	0.99	2.88	0.002	1.21
BG	Retrolenticular part of Internal Capsule (R)	1441	72	147	60	1.00	2.88	0.002	1.21
BG	Posterior Thalamic Radiation (L)	393	123	64	82	1.00	2.88	0.002	1.21
BG	Posterior Thalamic Radiation (L)	393	51	117	53	1.00	2.88	0.002	1.21
BG	Anterior Thalamic Radiation (L)	1345	116	66	90	1.00	2.88	0.002	1.21
BG	Anterior Thalamic Radiation (R)	2125	71	142	75	1.00	2.88	0.002	1.21
BG	Corticospinal Tract (L)	1218	116	91	100	1.00	2.88	0.002	1.21
BG	Corticospinal Tract (R)	2052	63	107	93	1.00	2.88	0.002	1.21
T	Inferior Fronto-Occipital Fasciculus (L)	1059	123	65	83	1.00	2.88	0.002	1.21
T	Inferior Fronto-Occipital Fasciculus (R)	4340	69	141	59	1.00	2.88	0.002	1.21
F	Uncinate Fasciculus (L)	144	109	144	100	1.00	2.88	0.002	1.21
F	Uncinate Fasciculus (R)	752	56	127	59	1.00	2.88	0.002	1.21
	***Axial Diffusivity***								
	**S>HC**								
BG	Anterior thalamic radiation (R)	322	66	117	106	0.99	2.05	0.02	0.88
BG	Corticospinal Tract (R)	433	65	113	105	0.99	2.05	0.02	0.88
T	Inferior fronto occipital fasciculus (R)	342	55	75	83	0.99	2.05	0.02	0.88

Abbreviations: FA = Fractional Anisotropy; RD = Radial Diffusivity; AD = Axial Diffusivity; S = Stroke group; HC = Healthy Control group; BG = Basal Ganglia; T = Temporal; F = Frontal; L = Left; R = Right.

**Table 5:** Clusters of significant FA and RD differences between Stroke groups with good and poor recovery and Healthy Controls group.

Brain Lobe	Anatomical Region	Size (mm^3^)	MNI coordinates			Z-max	Z	p	d
			x	y	z				
	***Fractional Anisotropy***								
	**HC>SG**								
F	Genu of Corpus Callosum (L)	231	91	110	97	0.99	2.05	0.02	0.88
	**HC>SP**								
BG	Pontine Crossing Tract (L)	194	94	149	85	0.99	2.05	0.02	0.88
F	Genu of Corpus Callosum (L)	1034	96	144	90	0.99	2.05	0.02	0.88
	Body of Corpus Callosum (L)	555	99	94	94	0.99	2.05	0.02	0.88
BG	Corona Radiata (L)	1180	109	140	101	1.00	2.05	0.02	0.88
BG	Posterior Thalamic Radiation (L)	215	116	68	90	0.99	2.05	0.02	0.88
BG	External Capsule (L)	661	123	119	74	0.99	2.05	0.02	0.88
BG	Anterior Thalamic Radiation (L)	347	113	158	90	0.99	2.05	0.02	0.88
BG	Corticospinal Tract (L)	726	117	107	97	0.99	2.05	0.02	0.88
P	Cingulum (cingulate gyrus) (L)	284	100	148	90	0.99	2.05	0.02	0.88
P	Forceps major (L)	382	116	68	90	0.99	2.05	0.02	0.88
P	Forceps minor (L)	326	94	149	85	0.99	2.05	0.02	0.88
F	Inferior fronto occipital fasciculus (L)	414	110	148	98	1.00	2.05	0.02	0.88
T	Inferior longitudinal fasciculus (L)	133	117	68	91	0.99	2.05	0.02	0.88
F	Superior longitudinal fasciculus (L)	663	111	141	101	1.00	2.05	0.02	0.88
F	Uncinate fasciculus (L)	145	112	160	97	0.99	2.05	0.02	0.88
	***Radial Diffusivity***								
	**SP>HC**								
BG	Pontine crossing tract (L)	176	94	149	85	1.00	2.33	0.01	0.98
F	Genu of corpus callosum (L)	768	96	145	90	1.00	2.33	0.01	0.98
	Body of corpus callosum (L)	306	115	71	90	0.99	2.33	0.01	0.98
BG	Anterior limb of Internal Capsule (L)	376	100	132	70	0.99	2.33	0.01	0.98
BG	Corona radiata (L)	1551	109	140	101	1.00	2.33	0.01	0.98
BG	External capsule (L)	417	123	130	69	1.00	2.33	0.01	0.98
BG	Anterior thalamic radiation (L)	890	111	158	88	1.00	2.33	0.01	0.98
BG	Corticospinal tract (L)	460	118	110	91	1.00	2.33	0.01	0.98
P	Cingulum (cingulate gyrus) (L)	174	99	148	90	1.00	2.33	0.01	0.98
P	Forceps major (L)	165	116	68	90	0.99	2.33	0.01	0.98
P	Forceps minor (L)	247	94	149	85	1.00	2.33	0.01	0.98
F	Inferior fronto-occipital fasciculus (L)	274	110	147	98	1.00	2.33	0.01	0.98
F	Superior longitudinal fasciculus (L)	375	112	140	101	1.00	2.33	0.01	0.98

Abbreviations: SG = Stroke group with good recovery; SP = Stroke group with poor recovery; HC = Healthy Control group; BG = Basal Ganglia; T = Temporal; F = Frontal; P = Parietal; L = Left.

### 3.4. Axial and Radial Diffusivity

**Figure 2:**
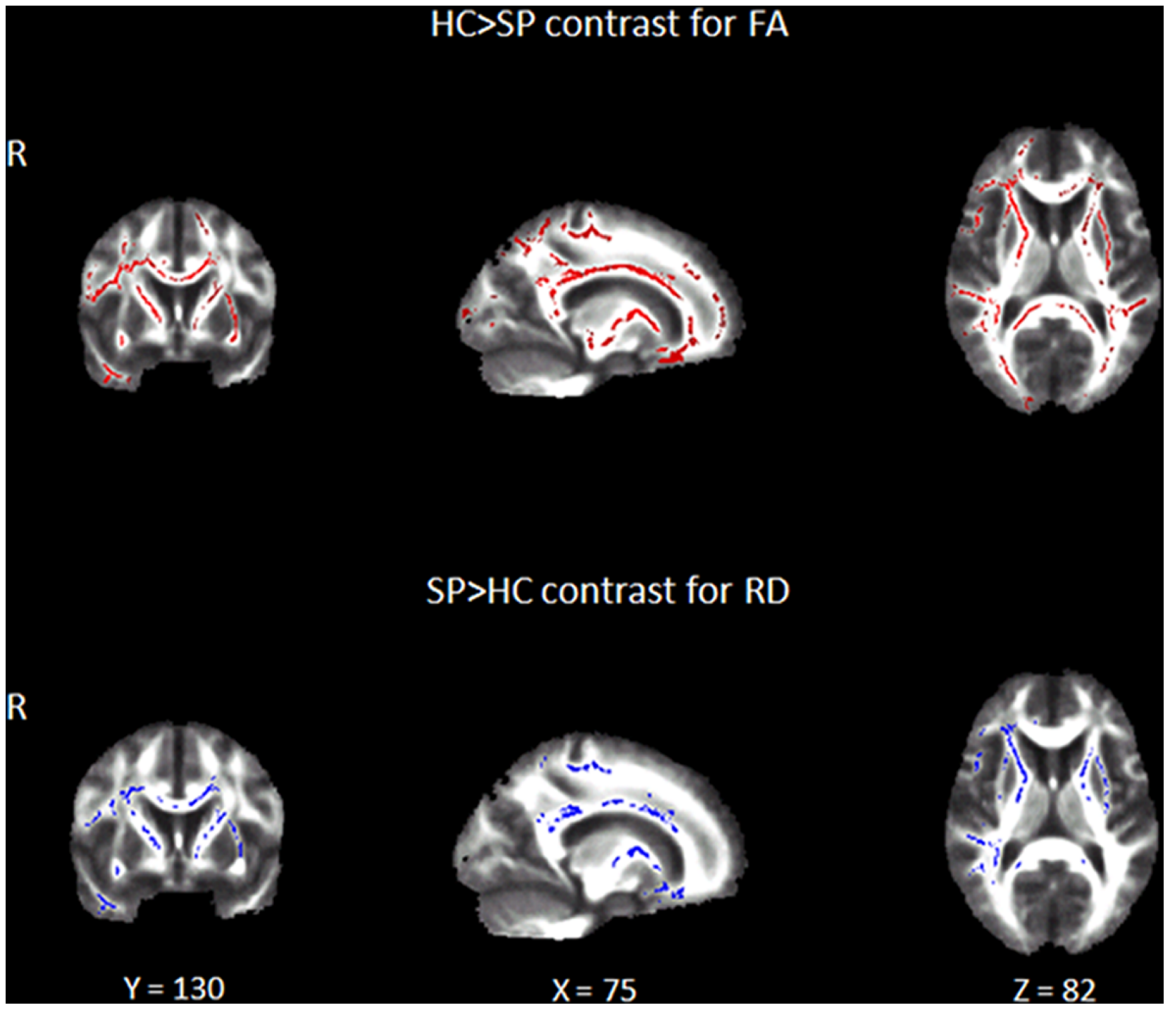
Significant changes in FA and RD for the stroke group with poor recovery at 3 months when compared with healthy controls. The red and blue colors show clusters of significant decrease of FA and increase of RD. Statistical maps are represented in radiological convention (right corresponds to left hemisphere) and are displayed superimposed on an MNI152 template. The threshold for significance was set at p≤0.02 corrected for multiple comparisons.

To investigate potential mechanisms underlying WM changes in stroke patients, both AD (λ|| = λ1) and RD [λ⊥ = (λ2+λ3)/2] maps were also analyzed. We found significant increase in both AD and RD for the S group (p = 0.002, d = 1.21 for RD; p = 0.02, d = 0.88 for AD) relative to HC group (Table 4). When analyzed separately, SP group showed significant increase of RD in the same anatomical areas where this group had shown significant FA decrease (p = 0.01, d = 0.98 for all the regions). The SG group did not show any significant change in either RD or AD in any region (Figure 2, Table 5).

### 3.5. Relationship between White Matter Integrity and Neuropsychological Function for the Stroke Subgroups

To assess if cognitive performance was associated with WM disruption, a whole-brain ANCOVA was performed using the WM skeleton given by the FA as dependent variable and the scores of the relevant cognitive tests as predictors, with diabetes mellitus as covariate of no interest. We regressed out the effect of diabetes because previous studies have shown that directly affects white and gray matter structures [64]–[67]. Clusters showing a significant correlation between cognitive test scores and FA are summarized in table 6. The SG group showed significant positive correlation between FA values located in the right retrolenticular part of the internal capsule (rIC) and scores in the Semantic Fluency test. Negative correlations were found between FA values located in the left hemisphere and the time spent to complete the GPT and the TMT part –A. For the GPT, the most significant areas were the left superior corona radiata (SCR) (p = 0.015) and the left anterior thalamic radiation (ATR) (p = 0.02). For the TMT part –A, the most significant areas were the left SCR (p = 0.008), the right inferior fronto-occipital fasciculus (IFOF) (p = 0.019) and the left IFOF (p = 0.021). The SP group showed negative correlations between the time spent to complete GPT and FA values in the right inferior longitudinal fasciculus (ILF) (p = 0.034) and the right posterior corona radiata (PCR) (p = 0.045).

**Table 6:** Clusters of significant negative (−) and positive (+) FA correlations between the different stroke groups and performance in the Semantic, Trail Making part-A and Grooved Pegboard Test.

Brain Lobe	Anatomical Region	Size (mm^3^)	MNI coordinates			Z	p
			x	y	z		
	**Semantic Fluency Test**						
	***+SG group***						
Basal Ganglia	Retrolenticular part of IC (R)	3256	52	91	74	2.014	0.022
	**Trail Making Test (part A)**						
	***−SG group***						
F	Superior Corona Radiata (L)	15788	108	126	116	2.409	0.008
F	Inferior Fronto-Occipital Fasciculus (R)	7663	64	77	93	2.075	0.019
F	Inferior Fronto-Occipital Fasciculus (L)	4035	120	161	75	2.033	0.021
F	Superior Longitudinal Fasciculus (L)	264	124	121	93	1.995	0.023
P	Cingulum (L)	149	98	126	112	1.685	0.046
P	Forceps Major (L)	120	101	45	90	1.655	0.049
	**Grooved Pegboard Test**						
	***−SG group***						
F	Superior Corona Radiata (L)	27662	108	123	112	2.170	0.015
Basal Ganglia	Anterior Thalamic Radiation (L)	7188	108	126	114	2.054	0.02
P	Splenium of Corpus Callosum (L)	116	111	76	94	1.695	0.045
Basal Ganglia	Posterior Thalamic Radiation (L)	390	120	57	73	1.685	0.046
	−***SP group***						
T	Inferior Longitudinal Fasciculus (R)	917	46	101	74	1.825	0.034
F	Posterior Corona Radiata (R)	136	62	87	94	1.695	0.045

Abbreviations: F = Frontal, P = Parietal; T = Temporal; R = Right; L = Left.

Between-group comparisons showed that correlation with semantic fluency scores was significantly stronger for the SP than for the SG in the right rIC (p<0.001). It was also stronger for the SP than for the HC in the right posterior thalamic radiation (pTR).

## Discussion

One of the most important clinical questions after stroke is patient’s potential for recovery from stroke-induced deficits. This question is of considerable interest given the impact of WM abnormalities for cognitive decline and the development of dementia after stroke [68]. WM changes after stroke are important determinants for presentation and severity of the neurological deficits as well as for prospects of recovery or secondary cognitive decline.

The present study aims to identify the effects of right hemispheric stroke on patient's cognitive recovery at three months after stroke. As an extension of previous studies, we were focusing on the left (contralesional) hemispheric WM. It has been reported that focal cerebral infarcts can lead to tissue alterations in remote connected regions, which are related to wallerian degeneration and cortical deafferentation. These secondary degenerative processes, usually detected within the ipsilateral hemisphere [69], can lead to a progressive atrophy [70], and they have been related to stroke recovery.

The role of the non-injured hemisphere in stroke recovery, however, is still controversial. In the contralesional hemisphere, only functional abnormalities have been identified in humans. Some imaging studies suggest that contralesional functional networks are significantly involved in post-stroke functional recovery [33], [71]; although the interpretation of the results regarding to their positive or negative implication in patient’s recovery is disputed (for a review of the literature see [71]). From a structural point of view, some DTI studies reported increased anisotropy in the contralesional hemisphere (e.g. thalamus) after stroke [24], [25], structural remodeling in ipsilesional and contralesional corticospinal tracts [25], and changes in the number of neural pathways in both ipsilateral and contralateral areas [34]. Studies of tract FA asymmetries have concluded that the contralesional corticospinal tract may play a role in motor recovery after unilateral stroke [72], [73], [74], [23].

In agreement with previous studies, we demonstrated that WM integrity (i.e. FA) was affected in the contralesional as well as in the ipsilesional hemisphere. Our findings also indicate that WM disruption is caused by demyelination rather than by axonal degeneration, as shown by the fact that the RD increase is more widespread than the AD increase. Axial and Radial components of the DTI tensor have been proposed as biomarkers of the type of neuronal damage [75], [76]: AD measures diffusivity in the principal diffusion direction, and it is proposed as a biomarker of axonal damage [77], [78], while RD is the average of diffusivities perpendicular to the principal direction of the tensor, and it is assumed to give information on the degree of demyelination [79], [80], [81].

In studies of small-vessel-disease both ischaemic demyelination and axonal loss have been found [82], [83]. Recently [84], RD was found to be the strongest predictor of executive dysfunction. This finding was interpreted in the sense that the ischaemic demyelination has greater influence than axonal degeneration on the presence of cognitive impairment, therefore it was proposed as a more reliable biomarker than AD. Our study not only provides some support for the role of demyelination in stroke patients at three months after suffering a stroke, but also provides a relationship between this event and the presence of a poorer cognitive performance.

The relevance of these changes is demonstrated by the fact that patients with poor cognitive recovery showed stronger WM disruption in the left hemisphere. The correlation of the WM changes with cognitive performance – especially in the contralesional hemisphere – further supports their functional importance. Notice that a higher score in SF and a lower score in TMTA and GPT means better cognitive performance. Therefore, the combination of a positive correlation with SF scores and a negative correlation with TMTA and GPT scores means a positive correlation with cognitive performance in general. It is important to mention that this correlation was stronger in SG patients than in SP patients. This finding can be explained by the more severe damage of WM in the SP group: comparing Table 5 and 6, it is obvious that most of the brain areas showing correlation in SG group are affected in the SP group. Although, changes of the contralesional hemisphere can be due both to the degenerative and protective processes (i.e. compensation), our findings correspond to the WM degeneration as confirmed by their disruptive nature (decreased WM integrity mostly due to demyelination), and their correlation with cognitive performance (i.e. lower WM integrity co-occur with worse performance).

Our findings are in agreement with other studies with stroke patients [25], [34] and extend our previous research with resting state [85] providing structural ground to the difficulty of SP patients to compensate their cognitive deficits after stroke.

SP patients showed significant deficits in attentional, motor, executive and processing speed functions when compared to HC. This profile has been related to vascular lesions in brain structures harboring frontal-subcortical circuits [86], something which is frequent in strokes that affect the vascular territory supplied by the MCA. Moreover, SP patients showed lower FA values in major left and right WM tracts that run along the anterior-posterior axis of the brain, supporting fronto-posterior and fronto-subcortical network interactions. These networks have been associated with executive functions [87]. Furthermore, WM disruption in the Body of Corpus Callosum for the SP group supports the suggestion made by Meguro et al (2000) who counted structural disruption of the corpus callosum as a sign of existing changes in the non-injured hemisphere. The relationships between structural changes reported here along with our previous findings imply their importance in clinical recovery and emphasize that not only lesion volume or lesion localization but WM integrity of the non-lesioned hemisphere are also important determinants of post-stroke recovery.

The generalizability of our findings is restricted by our relatively low sample size. On the other hand, our sample was quite homogenous regarding the lesion (all right-sided, first-time infarct) and demographic characteristics (e.g. vascular risk factors).

## Conclusion

According to our knowledge, our study is the first characterizing WM changes in relation to cognitive recovery in patients at three months post stroke and matched healthy participants.

We have demonstrated not only the involvement of the contralesional hemisphere but also that its involvement correlates with cognitive recovery. The results reported in this paper broaden our view of the factors that may play a role in patient cognitive recovery after stroke. Future longitudinal studies may further improve our understanding of the evolution of poststroke changes in the contralesional WM microstructure and the relevance of the WM changes observed in this study.

Moreover, our results demonstrate that DTI provides information about the mechanism of WM pathology, and may help to explain apparent severity and cognitive outcomes. In the future, DTI may serve as a biomarker of cerebral plasticity and help evaluating a patient’s response to rehabilitation. Predicting which patients will have worse outcomes in the chronic phase is a pivotal question for restorative neurology and can help us to adjust rehabilitation therapies more efficiently to each patient’s needs.

Finally, taking into account that we assessed only stroke patients at 3 months following ischemic stroke, our results should be interpreted carefully. Inter-individual differences in brain structure might be the result of variations in life experience or of different genetic predispositions that should be take into account in future studies with large samples [88].
